# Association between Obesity and Oral Health Status in Schoolchildren: A Survey in Five Districts of West Bengal, India

**DOI:** 10.5005/jp-journals-10005-1517

**Published:** 2018-06-01

**Authors:** Sonali Halder, Rahul Kaul, Paras Angrish, Subrata Saha, Bhaswar Bhattacharya, Malay Mitra

**Affiliations:** 1 Assistant Professor, Department of Pedodontics and Preventive Dentistry Dr R Ahmed Dental College and Hospital, Kolkata, West Bengal India; 2 Postgraduate Student, Department of Pedodontics and Preventive Dentistry Dr R Ahmed Dental College and Hospital, Kolkata, West Bengal India; 3 Postgraduate Student, Department of Pedodontics and Preventive Dentistry Dr R Ahmed Dental College and Hospital, Kolkata, West Bengal India; 4 Professorm Department of Pedodontics and Preventive Dentistry Dr R Ahmed Dental College and Hospital, Kolkata, West Bengal India; 5 Associate Professor, Department of Pedodontics and Preventive Dentistry Dr R Ahmed Dental College and Hospital, Kolkata, West Bengal India; 6 Professor and Head, Department of Pedodontics and Preventive Dentistry Dr R Ahmed Dental College and Hospital, Kolkata, West Bengal India

**Keywords:** Body mass index, Dental caries, Obesity.

## Abstract

**Introduction:**

Childhood obesity, dental caries, and periodontal disease are major public health problems due to their adverse impact on the growth and development of children. Obesity and oral health are associated as both share some common risk factors.

**Aim:**

The specific aim of the study was to determine the correlation, if there is any, between obesity and oral health in children.

**Materials and methods:**

A cross-sectional survey was conducted in five districts of West Bengal, India. A total of 1,227 school-going children of 6 to 12 years were examined from the districts of Howrah, Hooghly, West Midnapore, South 24-parganas, and North 24-parganas. Following indices were analyzed: Decayed-missing-filled teeth (DMFT), decayed, extracted, filled teeth (deft), simplified oral hygiene index (OHI-S). Depending on their nutritional status, subjects were categorized as being normal weight, overweight, and obese. Logistic regression analysis were applied to the study to find out the association between the above-mentioned dental indices and independent variables: Gender and nutritional status.

**Result:**

A positive association was found between obesity and oral health status in children.

**Conclusion:**

Considering the results of this study, it was concluded that obesity is related to oral hygiene status of children. In order to improve oral hygiene status in children, dietary modifications need to be done.

**How to cite this article:** Halder S, Kaul R, Angrish P, Saha S, Bhattacharya B, Mitra M. Association between Obesity and Oral Health Status in Schoolchildren: A Survey in Five Districts of West Bengal, India. Int J Clin Pediatr Dent 2018;11(3):233-237.

## INTRODUCTION

“EAT TO LIVE, DON’T LIVE TO EAT” becomes an important thought of human life as obesity is increasing day by day. Obesity and oral health are correlated as both share some common risk factors like dietary, genetic, socioeconomic, and lifestyle issues.^1^ Because of its global distribution and severe consequences, which include type II diabetes, osteoporosis, hypertension, and cardiovascular diseases, obesity is a serious health threat.^2^ Lifestyle factors, such as lack of physical activity, changes in eating habits, and social changes, have been considered as crucial factors for the global spread of obesity. Furthermore, dental caries and periodontal disease have traditionally been considered one of the greatest public health burdens because of their adverse impact on the growth and development of children.^3,4^ Oral health and obesity share common risk factors, as both are associated with unhealthy dietary habits—sugary soft drinks, snacks, and sugar-rich diets. Therefore, the World Health Organization (WHO) emphasizes the need to adopt a unified approach for the promotion of general and oral health instead of the previous single-level strategies.^4^ The “common risk factor” approach has been proposed as more rational, cost-effective, sustainable, and affordable.^4^ The period from childhood to adolescence is a critical stage of life during which children acquire important behavioral habits relevant to their general and oral health.^5^ Unhealthy dietary habits established in childhood tend to be carried throughout adulthood. Increased urbanization and economic development in most countries have led to adverse dietary change toward higher consumption of sugar and fat and reduced the intake of dietary fiber and this impairs oral health as well as increases the body mass index (BMI).^6^ Obesity is abnormal or excessive fat accumulation that may impair health.^7^

Etiologically sugar (sucrose) and some other carbohydrates are well documented to be the main dietary culprits in dental caries. Per capita sugar consumption directly varies with dental caries prevalence in any nation which has been observed during World War II.^8^ An association may exist between dental caries and obesity particularly in children, as they can withstand sweets more than the adult, the “Bliss Effect.”^9^ To measure the intensity or extent of dental caries in terms the number of teeth WHO recommends DMFT/deft index.

## AIM

The aim of the study was to determine the correlation, if there is any, between obesity and oral health in schoolchildren.

## MATERIALS AND METHODS

### Inclusion Criteria

A total of 1,227 school-going children of 6 to 12 years participated from five districts of West Bengal, namely Howrah, Hooghly, 24-Parganas north, 24 Parganas south, and Midnapore (West).

### Exclusion Criteria

- Children aged less than 6 years and more than 12 years
- Children with any congenital defects
- Children with persistent medical problems (continuous use of sugar-containing drug is a compulsion)
- Children with defects of enamel /dentin other than dental caries
- History of any periodontal treatment within last 6 months

The study design was placed before ethical committee of Dr R Ahmed Dental College and Hospital, Kolkata for permission to undertake the study.

Each selected school was contacted individually to obtain permission of the Head of the Institution after clarifying the aim of the study. Nature and purpose of the study were explained to parents in their preferred language. Its voluntary nature and strict confidentiality were assured. Parents were informed about the purpose of the study and they signed an informed consent form.

This cross-sectional study included 1,227 school-going children (767 males and 460 females) of age group 6 to 12 years. Stratified random sampling was done. Under the source of natural light in the room, dental caries was examined with mouth mirror and dental explorer. Dental caries was assessed as per the WHO Geneva Criteria 1997. To measure the intensity or extent of caries in terms of the number of teeth, WHO recommends DMFT/deft index. No radiograph was taken. Debris and calculus were examined by dental explorer and mouth mirror and assessed by OHI-S index according to Green and Vermillion (1964). The tooth was not cleaned of debris, nor was compressed air used, but where visibility was obscured by excess debris or moisture, this was removed with gauze or cotton roll.

The height and weight of participants dressed in light clothing without shoes were determined. Standing height was measured using a wall mounted stadiometer. The BMI was calculated as follows: BMI = weight in kg divided by height in meter square. The BMI was categorized according to WHO criteria (2004). Underweight was classified as BMI < 18.50, normal BMI 18.50-24.99, overweight >25.00, and obese >30 kg/m^2^.

Descriptive and interferential analysis was done for the data using Statistical Package for the Social Sciences version 19.0. The level of significance was set at ≤0.05.

## RESULTS

The cross-sectional study sample consisted of 1,227 children of age group 6 to 12 years, selected randomly from different private and government schools of five districts of West Bengal, according to the inclusion criteria.

For evaluation, the mean and standard deviation (SD) for the values of all the variables were computed and tabulated. Logistic regression analysis was applied to the study to find out association between the above-mentioned dental indices and independent variables: Gender and nutritional status. Chi-square test was done to find if any significant statistical correlation between two variables was there or not.

Table 1 shows the demographic characteristics of the study.

Table 2A shows the caries incidence in both males and females. The mean deft in females was 2.30, and in males, it was 2.16. The mean DMFT in females was 0.23, and in males, it was 0.22. The regression coefficient (b) for deft is 0.125 and that for DMFT is 0.003 (Table 2B).

Table 3 shows the distribution of females and males based on their BMI. In this study, 68% female children and 61% male children were underweight, whereas 10% in female and 26% in male were normal weight; 17% of female and 10% male were overweight; 5% female and 3% male were obese.

**Table 1:** **Table 1:** Gender-wise sample distribution

*Gender*		*Samples*		*Percentage*	
Female		460		37	
Male		767		63	
Total		1,227		100	

**Table 2A:** **Table 2A:** Caries incidence in male and female

		*Female*				*Male*				
*Caries*		*Mean*		*SD (+/-)*		*Mean*		*SD (+/-)*	
Deft		2.30		1.68		2.16		0.38	
DMFT		0.23		0.64		0.22		1.66	

**Table 2B:** **Table 2B:** Caries incidence in male and female

		*Gender (female = 1)*			
*Caries*		*p-value*		*Coefficient (b)*	
Deft		0.17		0.125	
DMFT		0.95		0.003	

**Table 3:** **Table 3:** Distribution of female and male based on their BMI

*Gender*		*Underweight (%)*		*Normal weight (%)*		*Overweight (%)*		*Obese (%)*	
Female		68		10		17		5	
Male		61		26		10		3	

**Table 4A:** **Table 4A:** Caries distribution in different BMI group

*Caries*		*Underweight*		*Normal weight*		*Overweight*		*Obese*	
Mean deft		1.80		2.15		4.01		3.50	
SD deft (+/-)		1.58		1.64		1.73		1.72	
Mean DMFT		0.09		0.26		0.69		0.64	
SD DMFT (+/-)		0.56		0.62		0.66		0.67	

**Table 4B:** **Table 4B:** Caries distribution in different BMI group

		*Body mass index*				*Gender (Female = 1)*			
*Caries*		*p-value*		*Coefficient*		*p-value*		*Coefficient*	
Deft		<0.01		0.114		0.17		0.125	
DMFT		<0.01		0.034		0.92		0.003	

**Table 5A:** **Table 5A:** Distribution of OHI-’S’ across different BMI group

*OHI-S*		*Underweight*		*Normal weight*		*Overweight*		*Obese*	
Female		0.55		0.62		1.07		0.99	
SD (+/-)		0.30		0.24		0.45		0.25	
Male		0.45		0.25		1.03		1.22	
SD (+/-)		0.35		0.29		0.36		0.48	

**Table 5B:** **Table 5B:** Distribution of OHI-’S’ across different BMI group

		*Body mass index*				*Gender (Female = 1)*			
*OHI-S*		*p-value*		*Coefficient (b)*		*p-value*		*Coefficient (b)*	
		<0.01		0.028		<0.01		0.189	

Caries distribution in different BMI group is analyzed in Table 4A. In underweight category, mean deft is 1.80 and mean DMFT, 0.09. Mean deft in normal weight category is 2.15, and DMFT is 0.26. In overweight, mean deft is 4.01 and mean DMFT is 0.69. Obese students have 3.50 of mean deft and 0.64 of mean DMFT. For both deft and DMFT, p-value ≤0.01, regression coefficient (b) for deft = 0.114, and for DMFT = 0.034. In both cases, p-value was <0.01 which meant that BMI significantly influenced deft and DMFT as shown in Table 4B.

Table 5A shows the distribution of mean OHI-S in different BMI groups. Among male students, mean OHI-S was 0.45 in underweight, 0.25 in normal weight, 1.03 in overweight, and 1.22 in obese. In case of female students, the mean OHI-S was 0.55 in underweight, 0.62 in normal weight, 1.07 in overweight, and 0.99 in obese. The BMI influences OHI-S in positive direction; p-value < 0.01 means this relation is statistically significant, where regression coefficient (b) is 0.028 (Table 5B).

The results show that BMI is positively associated with the deft, DMFT, and OHI-S.

## DISCUSSION

Childhood obesity and dental caries are both ubiquitous. Increased calorie intake associated with sugars and other carbohydrates, especially when added with physical inactivity, has been implicated in childhood obesity.^10^ It has been characterized by energy and metabolism imbalance and is responsible for the onset of multiple health complications.^9^

The present study found a positive correlation between BMI and dental caries (p < 0.01, regression coefficient b = 0.114 for BMI and deft, p < 0.01 and b = 0.034 for BMI and DMFT. That is, caries incidence increased with increase in BMI. This was in accordance with studies conducted by Hong et al^12^ and Bailleul-Forestier et al.^13^ Hong et al^12^ conducted a study to determine the association between obesity and dental caries in 2- to 6-year-old US children where 1,507 children were examined. Statistically significant association between caries and obesity was found for 60-72 months age group.

Bailleul-Forestier et al^13^ found that the severely obese children (n = 16) had a high level of caries incidence. There was a significant association between BMI and DMFT indices (p = 0.01) in the obese group.

This observation supported the findings of Willer-shausen et al^14^ and Sadegi and Elizadeh^15^ where caries incidence was positively correlated with BMI.

Chen et al^16^ found a different result, but in their study, only 3-year-old children were examined and caries prevalence was not significantly correlated with BMI. At the age of 3, there are some important factors, such as bottlefeeding and there is still major parental control over it. It is not the time for children to eat away from home.

Diet and socioeconomic status (SES) is closely related to BMI and dental health. Level of parental education and family income influence the food choice via availability of the resources to purchase a higher quality of food and awareness for nutritious alternatives.

For a low-income family, price takes a larger role than taste and quality of food. Young people from lower SES consume cheaper snacks and sweets and eat less fruit and vegetables than the higher socioeconomic group (Van der Horst 2007 and Sausenthaler 2007),^17^ which leads to high BMI as well as dental caries.

Diet plays an important role in developing both dental caries and obesity. Diet refers to the customary allowance of food and drink taken by any person from day to day and nutrition concerns the effect of diet on metabolic process of the body.^18^ Thus, the diet may exert an effect on caries locally and obesity generally. In relation to dental caries, it is important to emphasize the character of diet and number of times consumed rather than quantity.^19^

Honne et al^20^ found a positive correlation among obesity/overweight status and dental caries. But they considered diet as a risk factor for both obesity and dental caries in adolescents of Udupi district, South India. There was a significant difference in the frequency of sugar consumption between the BMI groups. Obese group of children had more caries than the overweight and low-normal-weight children. There was an association between caries experience and sugar consumption more than once/ day (odds ratio = 3.13, confidence interval = 1.25-7.85).

Soft drink consumption was the leading predictor for both dental caries and BMI in the study of Khadri et al.^21^ He investigated the relationship between oral health and obesity in adolescents in Sharjah city, United Arab Emirates. Analysis of the final model suggests that there is no clear relationship between obesity and dental caries, but that the consumption of soft drinks was a leading predictor for both.

Ethnicity and racial variations also influence the diet pattern and consequently obesity and oral health. Kranz et al^22^ stated that certain ethnic groups have better quality of diet than others, mainly because of their cultural background.

In the study of Khadri et al^21^ evaluating the role of sociodemographic indicators, BMI was shown to be correlated with respect to ethnicity, Arabs had marginally higher BMI in comparison with other nationalities in United Arab Emirates.

Physical inactivity is directly linked with high BMI. With the advent of advanced technologies, children prefer indoor activities, such as television watching, and playing computer and video games. Increased calorie intake with sugar and other carbohydrates, when added with physical inactivity, leads to obesity and dental caries. In the study of Khadri et al,^21^ the relationship between obesity and level of physical activity showed that physical inactivity was more prevalent among obese.

American Physical Activity Guideline, Australian Physical Activity Guideline, American Academy of Pediatrics, WHO, and International Obesity Task Force—all recommended restriction of sedentary activity to not more than 2 hours per day.

The factors like SES, diet, race, and ethnicity are not considered in this study. Also the study period is limited to collect the data from a larger sample size. Despite all limitations, it is evident from the present study that there is a positive correlation between obesity and oral health status. They have common risk factors and require a comprehensive multidisciplinary approach to pediatric patients by both medical and dental health care professionals.

## CONCLUSION

Mean DMFT score was lower than mean deft. The DMFT in underweight was 0.09, in normal weight 0.26, in overweight 0.69, and in obese 0.64, whereas deft was 1.80, 2.15, 4.01, and 3.50 respectively.

There is a statistically significant positive correlation between BMI with DMFT (r = 0.034) and BMI with deft (r = 0.114) (p <0.01) for both.

The deft and DMFT value did not have any significant correlation with the gender variation (r = 0.125) for deft, and (0.003) for DMFT, (p = 0.17) for deft, and (0.95) for DMFT.

Another cofactor considered for oral health status was OHI-S. In females, it was 0.55 in underweight, 0.62 in normal weight, 1.07 in overweight, and 0.99 in obese. In males, it was 0.45, 0.25, 1.03, and 1.22 respectively. There was a statistically significant (p <0.01) positive correlation between BMI and OHI-S (r = 0.028). The value of OHI-S varied significantly (r = 0.189 and p<0.01) with gender variation.

Overall, the association between obesity and oral health status was found positive. Considering the limitations of the present study, a positive association is found to exist between oral health status and obesity.

As our results indicate, being at risk of overweight or overweight is a potential risk factor for developing periodontal disease; promotion of healthy nutrition and physical activity in children and adolescents are necessary to prevent oral pathology, which can further affect the general health and well-being.
